# Climate change and C_4_ and C_3_ grasses in a midlatitude dryland steppe

**DOI:** 10.1002/ece3.70103

**Published:** 2024-08-01

**Authors:** Robert C. Anderson, Trace E. Martyn, Rachel R. Renne, Ingrid C. Burke, William K. Lauenroth

**Affiliations:** ^1^ Yale School of the Environment New Haven Connecticut USA; ^2^ Oregon State University La Grande Oregon USA

**Keywords:** big sagebrush, climate change, functional types, Green River Basin, perennial grasses, Wyoming

## Abstract

Climate change is projected to alter the structure of plant communities due to increasing temperatures and changes to precipitation patterns, particularly in midlatitude dryland ecosystems. Modifications to climatic suitability may lead to major community changes such as altered dominant plant functional types. Previous studies have indicated that climatic suitability is likely to increase for C_4_ grasses and decrease for C_3_ grasses in the Western United States. However, if no C_4_ grass species currently exist to serve as a propagule source, expansion into areas of increased suitability will be limited. We conducted a field and modeling study in the Upper Green River Basin (UGRB) of Western Wyoming to determine if (1) C_4_ grasses are present to provide a propagule source and (2) C_4_ grasses are likely to increase in importance relative to C_3_ grasses due to climatic changes. We searched 44 sites for C_4_ grasses to establish presence, and modeled suitability at 35 sites using 17 Global Climate Models, two greenhouse gas Representative Concentration Pathways (RCPs; 4.5 and 8.5), and two time periods (mid‐ and late century; 2030–2060 and 2070–2099, respectively). We found C_4_ grasses at 10 of the 44 sites, indicating that there is a present propagule source. Our model projected increases in suitability for both C_3_ and C_4_ grasses across sites for all RCPs and time periods. In the mid‐century RCP 4.5 scenario, the C_3_ functional type increased in projected biomass in 29 of 35 sites, and the C_4_ type increased in 31 sites. In this scenario, C_3_ grasses increased in projected biomass by a median 4 g m^−2^ (5% change), and C_4_ grass biomass increased by a median 8 g m^−2^ (21% change). Our study suggests that climate change will increase climatic suitability for grasses across the UGRB, and that all requirements are in place for C_4_ grasses to increase in abundance.

## Graphical abstract

Climate change will impact the distribution and structure of functional types in plant communities in drylands. We confirmed the presence of a C4 grass at the edge of its range in Western Wyoming and modeled the effect of climate change on functional types in the region. We found that while all functional types are projected to increase in abundance, C4 grasses will increase more than C3 grasses.

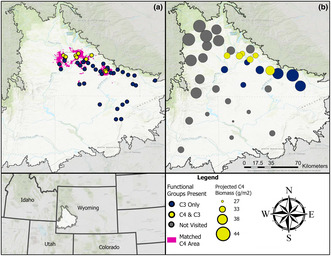

## 1 INTRODUCTION

Vegetation distribution at the global scale is primarily driven by climatic factors, specifically those related to seasonality and magnitude of temperature and precipitation (Huang et al., 2021). This is especially true in drylands, which are characterized by limited soil water availability as a result of low and variable precipitation and high evaporative demand (Noy‐Meir, 1973; Sala et al., 1997). As a result of this water resource limitation, drylands are more susceptible to the effects of climate change such as long‐term drought and higher levels of warming than humid areas (J. Huang et al., 2017; McCluney et al., 2012). These areas of high evaporative demand also run the risk of conditions exceeding the limits of plant ecophysiological stress, resulting in large mortality events (Renne et al., 2019). Thus, vegetation patterns in drylands are especially vulnerable to climate change‐induced alterations such as decreased above‐ground plant biomass and shifts in plant community composition (Maestre et al., 2012).

Grouping species by form or photosynthetic strategy can provide insight into ecological patterns spanning ecosystems with different species compositions (Smith et al., 1997). For example, differences in plant functional type have been shown to regulate trace gas fluxes (Epstein et al., 1998), affect the hydrology of grasslands (Pyšek et al., 2012), influence the frequency and duration of drought effects (Wilson et al., 2018), impact habitat suitability for a variety of wildlife species (Wisdom et al., 2005), and help answer an array of other ecological questions (Epstein et al., 1997). To assess the impacts that climate change may have on plant communities and how such changes could alter the rest of the system, it will be necessary to understand how plant functional‐type composition may change at local scales (Maestre et al., 2012).

Two of the most important plant functional types in drylands in the context of climate change are C_3_ (cool‐season) and C_4_ (warm‐season) grasses, since their distributions are primarily driven by temperature (Epstein et al., 1997). The photosynthetic efficiency of C_4_ grasses under high temperatures is greater than that of C_3_ grasses (Pearcy & Ehleringer, 1984), so globally C_4_ grasses are most prominent in the tropical and subtropical grassland regions, as well as in the warmest portions of temperate grasslands (Edwards & Still, 2008; Still et al., 2003). Studies have examined these patterns in the temperate midlatitude drylands of Western North America and much of Argentina and Uruguay. In these areas, relative abundance of C_3_ and C_4_ grasses closely follows patterns of mean annual temperature (MAT), mean annual precipitation (MAP), and precipitation seasonality (Epstein et al., 1997, 2002; Paruelo & Lauenroth, 1996). Research considering the impacts of climate change on C_3_ and C_4_ functional types indicates that rising temperatures and altered precipitation regimes are likely to increase climatic suitability for C_4_ species while decreasing suitability for C_3_ species (Epstein et al., 1997, 2002; Havrilla et al., 2023; Palmquist et al., 2021; Paruelo & Lauenroth, 1996). Shifts in the prevalence of these functional types can have major implications for ecosystem functioning, including carbon cycling and storage of soil organic matter (Liang et al., 2021; Pendall et al., 2011), resource availability and nutrient intake throughout food webs (Warne et al., 2010), trace gas flux and nitrogen cycling (Epstein et al., 1998), and differing phenological responses of species (Castillioni et al., 2022; Hong et al., 2022). However, these studies have primarily been administered at regional scales, and there is little information on how local factors may influence how these broad projections might play out at smaller scales.

The goals of our study were to assess the potential impacts of climate change on plant community structure and to test whether the assumption that C_4_ plants will increase in abundance while C_3_ species decrease will hold at the local scale. While climatic suitability is a major controlling factor for potential plant spread, it is not the only consideration (Young et al., 2022). Propagule availability from existing individuals is necessary for species to take advantage of increased climatic suitability (Lockwood et al., 2005; Young et al., 2022). In the Western United States, regional climatic suitability projections have been conducted (e.g., Palmquist et al., 2021), but realized changes at a local scale will depend on propagule availability of species found in a given area.

The Upper Green River Basin (UGRB) of Western Wyoming is a location where such a plant community shift is possible. The UGRB is dominated by the evergreen shrub big sagebrush (*Artemisia tridentata*) and currently supports primarily C_3_ perennial grasses but is projected to become much more suitable for C_4_ grasses under climate change (Palmquist et al., 2021). Little is known about whether or not C_4_ grasses are currently present in the UGRB, but the region is located adjacent to the range of *Bouteloua gracilis*, a C_4_ grass that is an important component of the vegetation throughout the eastern part of the state (Epstein et al., 1996; Lauenroth & Burke, 2008; Milchunas et al., 1989). It is widespread throughout the Great Plains (GBIF Backbone Taxonomy, 2023) and can compose as much as 90% of the plant basal cover in some areas (Milchunas et al., 1989). We used *B. gracilis* as the representative C_4_ grass functional type because of its relative dominance and more western distribution than other C_4_ grasses such as *B. dactyloides* (GBIF Backbone Taxonomy, 2023). These factors make *B. gracilis* the most likely C_4_ species to be found in the region and the most probable to have a large influence on the plant community. In a different locale, we may have had more or different C_4_ species to consider, but in the UGRB we were limited to *B. gracilis* to address our three questions: (1) What do we know about the current distribution of C_4_ grasses in the UGRB? (2) How will climate change impact climatic suitability for C_3_ and C_4_ grasses in the UGRB? (3) How might biomass for these functional types change relative to the other important functional types present in the UGRB from now until the end of the century?

To answer our first question, we performed an on‐the‐ground search for the C_4_ grass *Bouteloua gracilis*, informed by local knowledge and a site‐similarity matching script. We also collected soil samples at our field sites to obtain more information about the habitat suitability component of C_4_ distribution through the region. To answer questions 2 and 3, we used a process‐based plant community model to simulate plant biomass for the most important functional types at sites across the UGRB. To further explore the second and third questions, we interpolated site‐level plant biomass results from our model across the entire basin.

## 2 METHODS

### 2.1 Study area

Our research took place in the Upper Green River Basin (UGRB) of Western Wyoming during the summers of 2021 and 2022. We visited a total of 44 sites over these two summers. Sites primarily were located between Boulder, WY (42°44′50.6053″N, 109°43′13.6459″W) and Farson, WY (42°6′44.9514″N, 109°26′52.5495″W). The range of elevation across sites was 2031–2481 m, mean annual precipitation (MAP) ranged from 202 to 464 mm, and mean annual temperature (MAT) was between 2.2 and 4.2°C (PRISM Climate Group, Oregon State University, 2022). Precipitation levels are relatively consistent throughout the year, but snow is the most common form of winter precipitation in the region, while summer precipitation typically arrives as rain (Lauenroth et al., 2014; Schlaepfer et al., 2012; Zepner et al., 2021). This precipitation seasonality enables big sagebrush and other woody plants to dominate the vegetation (Lauenroth et al., 2014), with the understory comprising perennial grasses and forbs (Jordan et al., 2020). Bunchgrasses, primarily *Poa secunda*, *Hesperostipa comata*, and *Achnatherum hymenoides*, make up most of the understory biomass (Table 1). The UGRB is used by ungulates including cattle, mule deer, and pronghorn (Sawyer et al., 2005), and is important habitat for the imperiled greater sage‐grouse (Row et al., 2022).

**TABLE 1:** Species most commonly encountered during fieldwork in the Upper Green River Basin, listed alphabetically. Across life forms of the species found, all use the C_3_ photosynthetic pathway except for *Bouteloua gracilis*. This also denotes the species that is used as the respective functional type representative in the STEPWAT2 model for all results.

Form	Scientific name	Photosynthetic pathway	STEPWAT2 representative?
Shrub	*Artemisia tridentata*	C_3_	Yes
Shrub	*Chrysothamnus viscidiflorus*	C_3_	No
Grass	*Achnatherum hymenoides*	C_3_	No
Grass	*Elymus elymoides*	C_3_	No
Grass	*Hesperostipa comata*	C_3_	No
Grass	*Poa fendleriana*	C_3_	No
Grass	*Poa secunda*	C_3_	No
Grass	*Pseudoroegneria spicata*	C_3_	Yes
Grass	*Bouteloua gracilis*	C_4_	Yes
Forb	*Alyssum desertorum*	C_3_	No
Forb	*Eriogonum caespitosum*	C_3_	No
Forb	*Phlox hoodii*	C_3_	Yes
Forb	*Stenotus acaulis*	C_3_	No

### 2.2 Field approach

#### 2.2.1 Site selection

**FIGURE 1:**
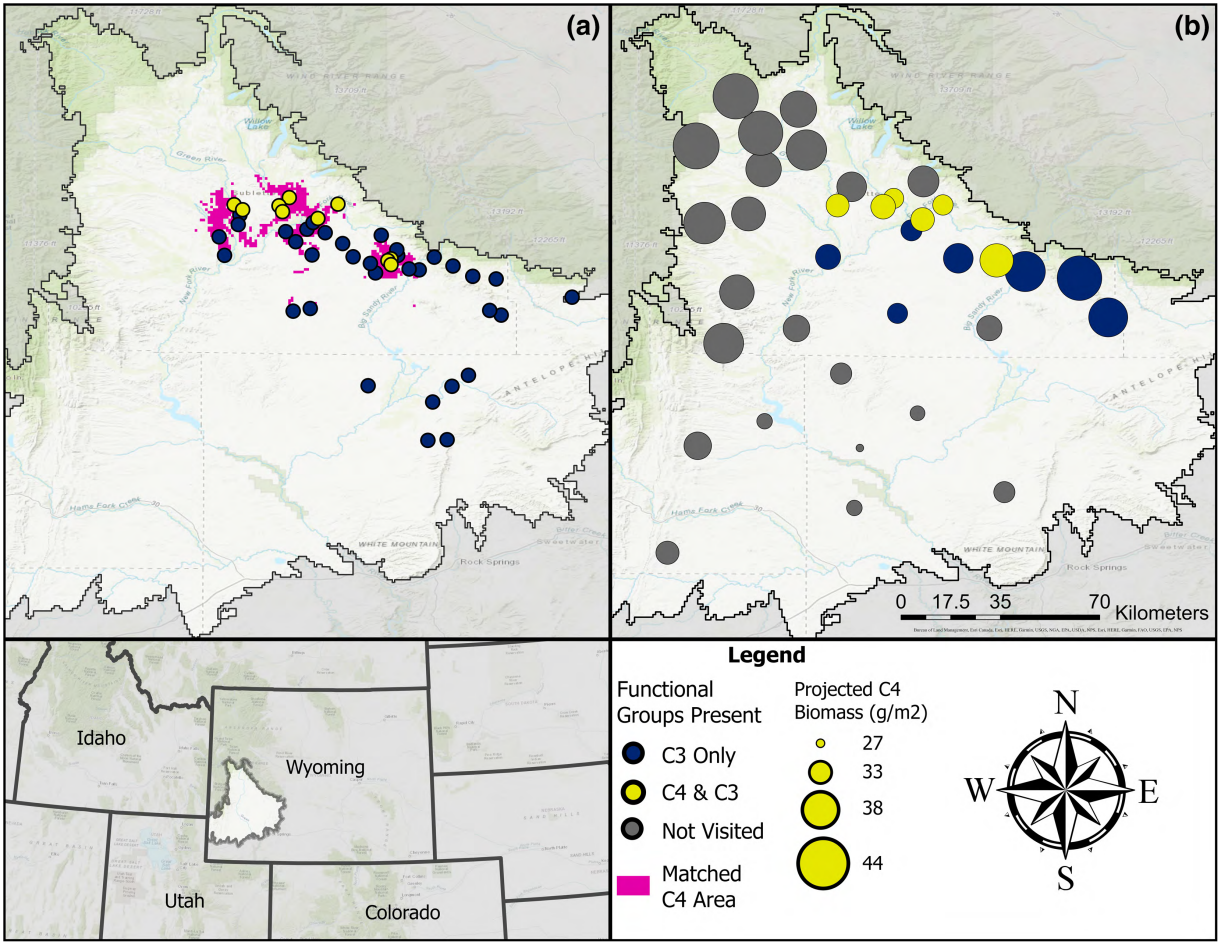
Locations of sites visited in the field (a) and sites used in the STEPWAT2 simulations (b). Blue points represent sites where C_4_ grasses were not found in the field, yellow points represent sites where C_4_ grasses were found in the field, and gray points represent sites not visited during fieldwork. In (a), pink areas are environmentally similar to sites containing C_4_ grass. In (b), point size represents the C_4_ grass biomass (in g m^−2^) projected at each site under the current (1990–2020) time period in STEPWAT2. Boundary indicates the portion of the UGRB designated as within our study area using environmental similarity (Renne et al., 2024).

We employed several strategies to select the 44 sites that we searched for the presence of the C_4_ grass, *Bouteloua gracilis* (Figure 1a). We randomly selected 20 sites, used locations of existing *B. gracilis* records to select 11 sites, and chose an additional 13 sites using a statistical algorithm to identify areas with similar environmental conditions to sites where we found *B. gracilis* using other methods.

To randomly select sites, we used maps from the US Bureau of Land Management (BLM) and a random number generator to choose latitude and longitude coordinates, rejecting sites that did not fall within BLM land. We used a combination of sources for records of *B. gracilis* presence, including our previous work in the area, herbarium records (RM Herbarium Specimen Search, 2021), and nine sites provided by local experts.

We selected all remaining sites by estimating the environmental similarity between the entire UGRB (at ~1 km resolution) and all sites known to contain *B. gracilis*. We used the rMultivariateMatching package as described in Renne et al. (2024) in R version 4.1.2 (R Core Team, 2021). We used the location of sites where we had identified *B. gracilis* in the UGRB prior to June 20, 2022 (*n* = 81) to identify the environmental conditions that favor this species within the study area. We used six key climatic variables based on Renne et al. (2024) and two soil texture variables: mean annual temperature, mean temperature in the warmest quarter, mean temperature in the coldest quarter, total annual precipitation, precipitation of the warmest quarter, precipitation of the coldest quarter, percent sand, and percent clay content of soils. A description of the bioclimatic variable quantities for the sites where *B. gracilis* was found is in Table S1. The rMultivariateMatching package contains ~1 km resolution raster data of bioclimatic variables (Hijmans et al., 2017) calculated from DayMet (Thornton et al., 2022) and soil texture variables, which were obtained from Soilgrids+ (Hengl et al., 2017), for the state of Wyoming. In our matching criteria, we prioritized sites with bioclimatic qualities within 5% of the range of values at our C_4_ sites (Table S1). We used the multivarmatch function in the rMultivariateMatching package to identify areas in the UGRB that were environmentally similar to sites known to support *B. gracilis*. The multivarmatch function produces an output raster of site similarity (calculated as a weighted Euclidean distance of the eight environmental variables). Similarity scores ranged from 0 to 773 (most to least similar), and we selected sites additional sites to search that had a similarity score of ≤10 with sites containing *B. gracilis*.

#### 2.2.2 Field search protocol

At each site we used a similar search effort to find *B. gracilis*. We spent between 1 and 3 h at each site conducting searches that covered between 2.4 and 4.8 km in distance. At sites where we found *B. gracilis*, we took GPS coordinates at each distinct patch. We defined a patch as an area containing *B. gracilis* that was over 10 meters away from the next patch. We measured the basal cover of 20 patches across the four sites where *B. gracilis* was found prior to June 20, 2022.

#### 2.2.3 Soil sampling

To quantify the soil texture requirements of habitat suitability for C_4_ grasses in the UGRB, we obtained soil samples from eight randomly selected field sites, four that contained C_4_ grasses and four that did not. We extracted soil samples using an auger at three depths: 0–10, 10–20, and 20–30 cm. We took three sets of samples at each site, dried them for 48 h, and processed them in the lab using the standardized modification of the Bouyoucos hydrometer method for texture analysis as described in Bouyoucos (1951). We then calculated the depth‐weighted average soil texture for each site from the soil layers using the equations found in Hengl et al. (2017) and performed a Welch two‐sample *t*‐test to compare the soil texture of sites with different functional compositions.

### 2.3 Modeling approach

#### 2.3.1 Model description

To model plant climatic suitability, we used the gap dynamics plant community model STEPWAT2 (Palmquist et al., 2018). STEPWAT2 integrates a stochastic, individual‐based plant simulation model (STEPPE) (Coffin & Lauenroth, 1990) with a deterministic, process‐based soil water balance model (SOILWAT2) (Schlaepfer et al., 2012). Figure S1, which displays the first figure from Palmquist et al. (2018), captures some of the sequence of events during the running of the model. SOILWAT2 runs on a daily time step, using weather, soil, and plant data to calculate the depth and temporal distribution of water available for the growth of each species or plant type. STEPPE runs on an annual time step and can simulate individual species or plant types, including information on root depth, distribution, and phenology. SOILWAT2 passes information of the sum (over depth and time) of available water for each plant type to STEPPE, which in turn uses this information to calculate the biomass increment of each individual of each species or plant type. Available water is allocated among individuals beginning with the largest and continuing in order of size until the amount for that type is exhausted. All individuals are vulnerable to mortality but those that did not get all of the water they needed for growth in a particular year have an increased probability of mortality. We ran our simulations with species representatives from the plant types of shrubs, C_3_ annual grasses, C_3_ perennial grasses, C_4_ perennial grasses, and forbs (Palmquist et al., 2018; Table 1). The perennial C_4_ grass in our simulations was *Bouteloua gracilis* (Table 1). We included different temperature response curves for C_3_ and C_4_ species and different water use efficiencies. More details about STEPWAT2 can be found in Palmquist et al. (2018, 2021).

We used STEPWAT2 to model 35 sites distributed across our study area. We selected 14 sites that were part of the 44 sites visited in the field, 17 randomly selected sites, and 4 sites used in Palmquist et al. (2021) (Figure 1b; Figure S2). For all 35 sites, we ran STEPWAT2 simulations for 300 years with 100 iterations using version 1.0.0 of the R program rSFSTEP2 (https://github.com/DrylandEcology/rSFSTEP2/releases/tag/v.1.0.0). Because STEPWAT2 does not start with any vegetation initialized, we used 300‐year simulations to ensure a steady state was reached. We only used the output of the final 100 years of simulation in our analysis, representing the period when the system had reached the steady state.

#### 2.3.2 STEPWAT2 simulations

We ran STEPWAT2 simulations for all sites under historical, current, and future climate conditions. We extracted current climate data from DayMet (Thornton et al., 2022) for the years 1990–2020. We derived future climate conditions from the Multivariate Adaptive Constructed Analogs (MACA) (Abatzoglou & Brown, 2012) downscaled Global Climate Models (GCMs) dataset using 17 GCMs under the greenhouse gas Representative Concentration Pathways (RCP) 4.5 and 8.5 for both mid‐century (2030–2060) and late century (2070–2099). We used these RCPs because they represent an intermediate climate change scenario and an extreme scenario (Shukla et al., 2022). We used historical data from MACA as well, which used the 17 GCMs to back project the climate in the time period 1950–1980. We included historical data to allow us to understand any climate change that occurred prior to our representation of current conditions. This resulted in a total of 86 unique climate scenarios run for each of the 35 sites, for a total of 3010 model runs. We included multiple GCMs to account for some of the uncertainty in future functional‐type biomass brought about by uncertainty in future climate projections. Past research has indicated that at least 13 models are required to capture 80% of the range in projections for 75% of Earth's surface, and this prediction ability increases with each additional model (McSweeney & Jones, 2016). For all our sites, we used a first‐order Markov weather generator to translate the daily weather projections in each time period into the 300‐year records required in the simulations (Palmquist et al., 2018, 2021). For use in the STEPWAT2 model, we obtained soil texture data for each site by downloading percentage of clay and sand from the USDA Web Soil Survey (Web Soil Survey, 2022). This source provides the soil composition by soil layer necessary for the model, and we used textures for eight layers: 0–10, 10–20, 20–30, 30–40, 40–60, 60–80, 80–100, 100–150 cm.

### 2.4 Data analysis

We conducted analyses using R version 4.1.2 (R Core Team, 2021). We used the ggplot2 package in R (Wickham, 2016) to create all plots. For spatial analyses and illustrations, we used ArcGIS Pro version 3.0.3 (ESRI, 2022). We report findings for RCP 4.5 since this is thought to be more representative of what is likely to happen compared to RCP 8.5 (Shukla et al., 2022). Results for RCP 8.5 are included in Supplementary Material S1.

#### 2.4.1 Changes in climate

To characterize future climate conditions, we averaged mean annual temperature and annual precipitation across the last 100 years of the simulation for each combination of site, RCP, GCM, and time period. We calculated the median and range of these variables across GCMs and sites (Table 2).

**TABLE 2:** Temperature and precipitation medians and ranges for historical (1950–1980), current (1990–2020), mid‐century (2030–2060), and late‐century (2070–2099) time periods. Values were estimated using the RCP 4.5 scenario and were calculated using a first‐order Markov weather generator. Medians and ranges are across 17 GCMs and 35 sites and were averaged across the final 100 years in the simulation.

Data source	Period	Temperature (°C)		Precipitation (mm)	
		Median	Range	Median	Range
MACA	Historical (1950–1980)	2.1	0.9–4.7	278	182–521
DayMet	Current (1990–2020)	3.1	2.0–5.3	303	182–614
MACA	Mid‐Century (2030–2060)	6.3	5.0–9.0	452	304–519
MACA	Late‐Century (2070–2099)	7.3	5.5–10.5	462	311–551

#### 2.4.2 Biomass trends

For each functional type, we averaged mean annual biomass over the last 100 simulation years of each combination of site, RCP, GCM, and time period. For each combination of site, RCP, GCM, and time period, we calculated the difference in future biomass of each functional type from its current value. We created heatmaps using R to assess the trends of projected differences by site and GCM under different scenarios for the grass functional types. We then calculated the number of sites increasing or decreasing in biomass based on the projected value under the median GCM. To remove the effect of extreme GCMs, we used the median for all biomass calculations. We created boxplots to illustrate the absolute and relative differences in simulated biomass for all functional types under historical, mid‐century, and late‐century time periods.

#### 2.4.3 Projected biomass interpolation

We used the rMultivariateMatching package (Renne et al., 2024) to create a raster of sites that most closely matched the environmental characteristics of all sites containing *B. gracilis* patches by the end of fieldwork in July 2023 (*n* = 125) (Table S2). Similar to our use of this package in the field search stage, we used eight key environmental variables to estimate similarity between sites where we observed *B. gracilis* and the entire UGRB (at ~1 km resolution).

We also used this process to determine an area for interpolation of our simulation output from the 35 STEPWAT2 modeled sites (i.e., we restricted our interpolated results to sites within the UGRB that were environmentally similar to our simulated sites based on similarity scores from the rMultivariateMatching package). This resulting area closely matched the distribution of potential big sagebrush habitat in the region (Renne et al., 2024), confirming its ability to be used as our area of interest. We used this extent layer, the STEPWAT2 projection results, and the interpolatePoints function within the rMultivariateMatching package to create maps of current and future changes in projected biomass extending through the region. We used the same variables as in other instances of using the rMultivariateMatching package, but rather than using the soil texture data from Soilgrids+ (Hengl et al., 2017), we used the percentage of clay and sand data from the USDA Web Soil Survey (Web Soil Survey, 2022) since that is what was used to incorporate soil layer data in our model. We calculated depth‐weighted average soil texture for each site from the soil layers obtained from the Web Soil Survey using the equation found in Hengl et al. (2017).

## 3 RESULTS

### 3.1 Field results

#### 3.1.1 Bouteloua gracilis distribution and habitat

We found only C_3_ grasses at 34 of the 44 sites we searched, while the other 10 contained both C_3_ and a C_4_ grass, *B. gracilis* (Figure 1a). These 10 sites contained a total of 125 patches of *B. gracilis*. The amount of *B. gracilis* was not evenly distributed among sites, with patch numbers per site ranging between 1 and 70 with a median of six patches per site. The measured patches of *B. gracilis* averaged 73 m^2^ (*n* = 20) basal area. Average measured patch size was also not evenly distributed, with means per site of 252 m^2^ (*n* = 3), 40 m^2^ (*n* = 11), 22 m^2^ (*n* = 4), and 13 m^2^ (*n* = 2), respectively. Analysis of areas that most closely matched the environmental conditions at the 10 sites where we detected *B. gracilis* indicated that 2% of the region has similar conditions (Figure 1a).

At the four sites with *B. gracilis* where we took soil samples, the average sand content was 80%, compared to 58% sand at the four sites containing only C_3_ grasses. The clay composition was 14% in the C_4_ sites and 27% in the C_3_ sites. The difference in composition between the two site types was significant for both components (*p* < .0005).

### 3.2 Model results

#### 3.2.1 Climatic variables across time periods

Across all sites and GCMs, projected median historical (1950–1980) temperature was 2.1*°*C, median current (1990–2020) temperature was 3.1*°*C, and projected future median temperature under RCP 4.5 was 6.3*°*C in the mid‐century (2030–2060) and 7.3*°*C in the late‐century (2070–2099) time periods. Median historical precipitation was 278 mm, median current precipitation was 303 mm, and projected future median precipitation under RCP 4.5 was 452 mm in the mid‐century and 462 mm in the late‐century time periods (Table 2).

#### 3.2.2 Functional type changes in biomass across time periods

Annual forbs and shrubs other than big sagebrush accounted for a very small proportion of community biomass and are therefore omitted here. Simulations under current climate conditions projected a median biomass of 36 g m^−2^ for C_4_ grasses, with a range of 27–44 g m^−2^ (Figure 1b). Of the simulation sites chosen to represent field sites containing C_4_ grass (6 of 35), five were in the bottom 50% of projected current C_4_ biomass. Median projected C_3_ grass current biomass was 67 g m^−2^ (range: 58–75 g m^−2^), median projected big sagebrush current biomass was 491 g m^−2^ (range of 377–845 g m^−2^), and median projected perennial forb current biomass was 18 g m^−2^ (range: 16–19 g m^−2^).

**FIGURE 2:**
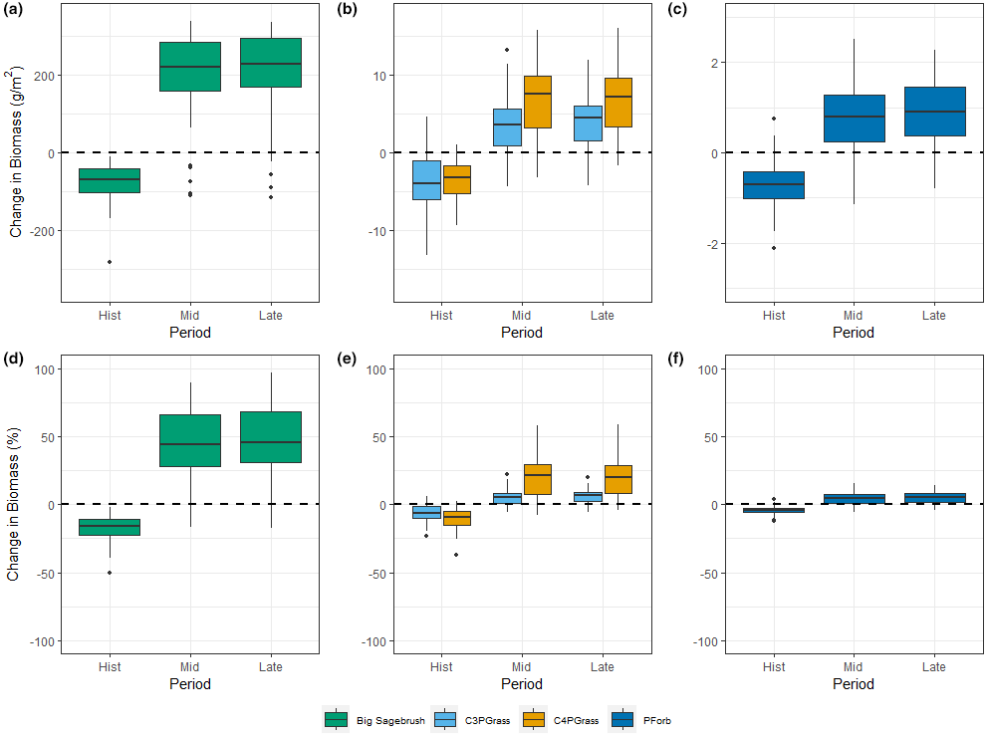
Changes in projected absolute biomass (g m^−2^) (a–c) and relative biomass (d–f) from the current (1990–2020) time period (0 line) to historical (1950–1980), mid‐century (2030–2060), and late‐century (2070–2099) periods. Biomass projections were averaged across the last 100 years of simulations; boxplots were created using values across all sites and GCMs for RCP 4.5. Plots are split into major functional groups based on the magnitude of absolute projected biomass difference: (a, d) Big Sagebrush; (b, e) C_3_ and C_4_ Perennial Grass; (c, f) Perennial Forbs. Note the differences in scale for the change in absolute biomass in each group.

Compared to current biomass projections, simulated median historical values for big sagebrush were 71 g m^−2^ lower (−16%) across all sites and GCMs (Figure 2a,d). Perennial C_3_ grass historical biomass was projected to be 4 g m^−2^ less (−6%) than current, and median perennial C_4_ historical biomass was 3 g m^−2^ less (−10%) (Figure 2b,e). Perennial forbs had 1 g m^−2^ less (−4%) projected biomass than in current conditions (Figure 2c,f).

Under RCP 4.5, median projected big sagebrush biomass increased by 220 g m^−2^ (44% increase) in the mid‐century and 231 g m^−2^ (46% increase) in the late century (Figure 2a,d). Perennial C_3_ grasses increased in median projected biomass by 3.6 g m^−2^ (5% increase) in mid‐century, and by 4.5 g m^−2^ (7% increase) in the late century. Perennial C_4_ grass projected biomass rose by a median of 8 g m^−2^ (21% increase) in mid‐century, but only 7 g m^−2^ (20% increase) in the late‐century period (Figure 2b,e). Perennial forb future biomass was projected to increase by median of 0.8 and 0.9 g m^−2^ (4% and 5% increase) for mid‐ and late‐century periods, respectively (Figure 2c,f). Biomass projections also increased across functional types and time periods under RCP 8.5, except for perennial forbs in the late‐century simulation, which saw minor decreases in projected biomass (Figure S3).

**FIGURE 3:**
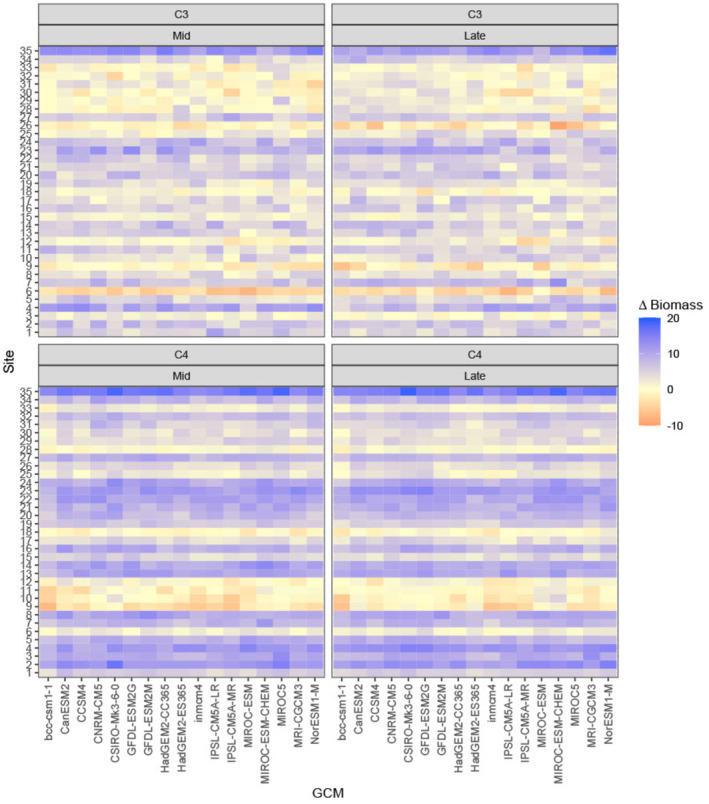
Heatmaps displaying the differences between biomass (g m^−2^) projections in mid‐century (2030–2060) and late‐century (2070–2099) time periods relative to current (1990–2020) for both C_3_ and C_4_ functional groups under RCP 4.5. Projected biomass differences are reported for each combination of the 35 sites and 17 GCMs used in the STEPWAT2 simulations.

Of our 35 modeled sites, C_3_ grasses increased in projected biomass at 29 sites under the mid‐century period for RCP 4.5, and C_4_ grasses projected biomass increased at 31 of the sites. In the late‐century period, the projected biomass of both functional groups increased at 32 of 35 sites (Figure 3). In RCP 4.5, there were no noticeable effects of GCM on projected biomass by site (Figure 3), but in RCP 8.5 some GCMs projected a similar change in biomass across all sites regardless of the site effect (Figure S4).

#### 3.2.3 Interpolating functional type biomass change through the UGRB

**FIGURE 4:**
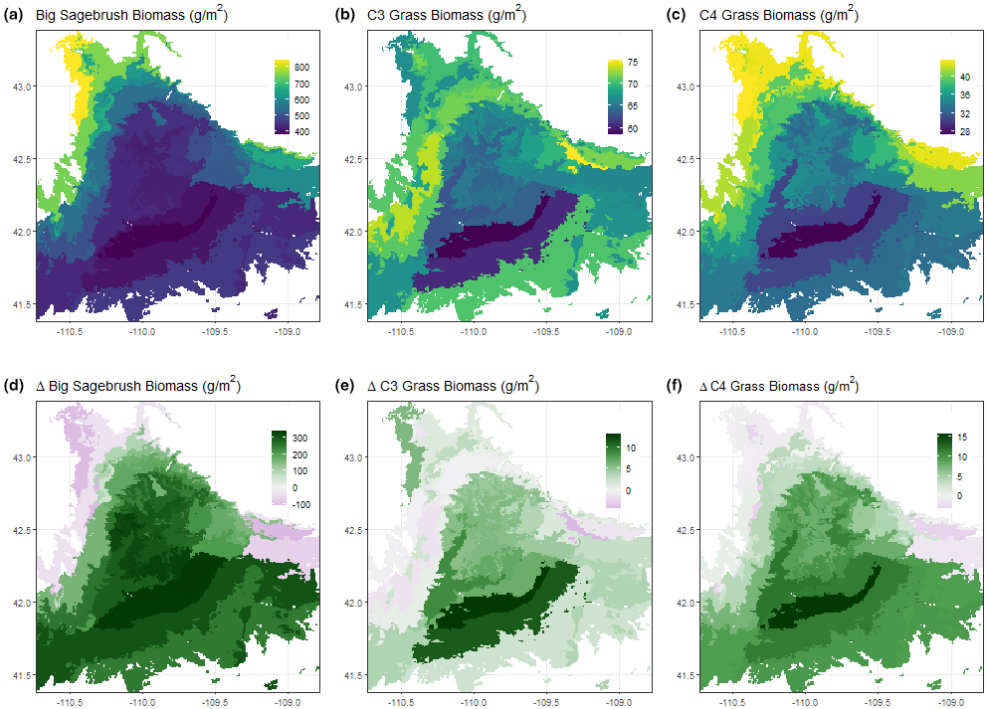
Projected biomass (g m^−2^) under current (1990–2020) climate conditions across the Upper Green River Basin for (a) Big Sagebrush, (b) C_3_ Perennial Grass, and (c) C_4_ Perennial Grass. Projected change in biomass (g m^−2^) from current to mid‐century (2030–2060) under RCP 4.5 for (d) Big Sagebrush, (e) C_3_ Perennial Grass, and (f) C_4_ Perennial Grass. Future projections represent the median of 17 GCMs used for each site.

Projected functional‐type biomass increased across most of the UGRB for all functional types under RCP 4.5 in the mid‐century. Big sagebrush projected biomass increased in 82% of the region (Figure 4a,d), C_3_ grass projected biomass increased in 86% of the basin (Figure 4b,e), and C_4_ grass projected biomass increased in 85% of the UGRB (Figure 4c,f). Perennial forb biomass increased across 87% of the region (Figure S5B,E).

## 4 DISCUSSION

We found that *Bouteloua gracilis* is present in the Upper Green River Basin of Western Wyoming, indicating that propagules are available to respond to future changes in climatic suitability for C_4_ grasses in the region. Our environmental similarity analysis and simulation modeling indicated that climatic suitability for the C_4_ functional type as represented by *B. gracilis* is currently high in the UGRB. Our simulation results suggest that climatic suitability for all functional types will increase across most of the basin with climate change, with C_4_ grasses representing the most substantial increase in herbaceous plants. Projected plant biomass increased across the majority of the UGRB by the middle of this century, and our results suggest that biomass may have already increased relative to the recent past. These results are consistent with other studies on plant range suitability with climate change in the region (Epstein et al., 2002; Martyn et al., 2023; Palmquist et al., 2021; Still & Richardson, 2015).

### 4.1 The variability of climatic change impact on plant communities at different scales

Throughout our study area, changing climate conditions followed the general trends that are predicted at the scale of the Western United States. Both mean annual temperature (MAT) and mean annual precipitation (MAP) are projected to increase in the mid‐ and late‐century time periods, and both have increased since the middle of the 20th century (Table 2). These trends hold at the scale of the entire big sagebrush range, with some variability in averages and seasonality of precipitation (Havrilla et al., 2023).

Accompanying these climatic conditions, we expect to see changes in the functional structure of the plant communities (Lajeunesse & Fourcade, 2023). Past studies have shown that the distribution of C_3_ grasses is negatively related to increasing MAT, while C_4_ productivity is positively related to MAT and MAP (Epstein et al., 1997; Paruelo & Lauenroth, 1996). Further, regional modeling studies have indicated that C_4_ grasses are likely to increase in abundance and productivity as temperature increases and precipitation regimes change, largely to the detriment of C_3_ grasses (Epstein et al., 2002; Havrilla et al., 2023; Palmquist et al., 2018, 2021; Winslow et al., 2003). However, recent studies of potential climate change impacts on *B. gracilis* are mixed, suggesting that *B. gracilis* is not likely to change its distribution much as a result of climate change (Avendaño‐González & Siqueiros, 2021), or that climate change will result in more range lost for *B. gracilis* than gained (Havrilla et al., 2023). Precipitation, and especially precipitation seasonality, is particularly important in controlling communities of C_3_ and C_4_ grasses (Avendaño‐González & Siqueiros, 2021; Epstein et al., 1997; Hutchinson & Schuman, 2002; Winslow et al., 2003). Precipitation events are expected to be more variable as the climate changes, which increases the difficulty of predicting community changes at the large scale (Fust & Schlecht, 2022; Gibson et al., 2019; Shukla et al., 2022). Despite the importance of assessing environmental and ecological changes over large areas, sub‐regional and local differences in climatic conditions mean that regional‐scale patterns of both climate and community changes may not hold locally (Havrilla et al., 2023; Lajeunesse & Fourcade, 2023; Maestre et al., 2012; Still & Richardson, 2015). Thus, our work provides insight into plant community changes at a local scale.

### 4.2 Expanding climatic suitability to future community composition through local propagule presence and habitat suitability

Another major consideration in assessing local‐scale community response to climate change is the availability of species to take advantage of increased climatic suitability. A range shift requires migration of species from their current locations to future suitable areas. Even if suitability is projected to increase, poor connectivity between propagule sources and new habitat, as well as dispersal limitation, will reduce the ability of a particular species to track climatic suitability, especially over short timeframes (Mallen‐Cooper et al., 2023; Raw et al., 2023). Our field study confirmed that the C_4_ grass *B. gracilis* is present in the UGRB, suggesting that these plants could serve as a propagule source for expansion of this species under projected climate change. It is especially important to have confirmed presence of C_4_ grasses due to the location of the UGRB, which is nestled between two mountain ranges. Even though *B. gracilis* is relatively widespread through much of Eastern and Central Wyoming (RM Herbarium Specimen Search, 2021), mountains can act as barriers to the genetic flow between populations of this species (Avendaño‐González et al., 2019). By finding this C_4_ species in the UGRB, we present evidence that this functional type may have the potential to respond to changing climatic suitability.

In addition to simply confirming the presence of *B. gracilis* in the UGRB, our work has also contributed to the knowledge of the distribution of this species. Of the 44 field sites we surveyed, we detected B. *gracilis* at 10, with a total of 125 individual patches found across the UGRB (Figure 1; Table S2). This increases our understanding of the distribution of *B. gracilis* in the UGRB; previously there was only one herbarium record from 1995 (RM Herbarium Specimen Search, 2021).

Consistent with previous research, our simulation modeling indicated that climatic suitability will improve for C_4_ grasses in the UGRB (Figure 3; Palmquist et al., 2018, 2021; Havrilla et al., 2023), with increases in C_4_ biomass across GCMs by the middle of the century for both RCP 4.5 and 8.5 for nearly all of our sites (Figure 3, Figure S4). It should be noted that our model also predicted higher levels of C_4_ biomass present through the UGRB than we found in the field (Figure 1). This may be partially due to several factors, including our inability to search all sites in the field, the possibility that C_4_ grasses have not yet spread to all suitable areas, or that current C_3_ species are limiting C_4_ grass spread. This also could be due to differences in soil texture across sites. Our field soil samples indicated that *B. gracilis* was found at sites with sandier soils than areas where it was not detected, a pattern also displayed with the sites used in our modeling; C_4_ sites had an average soil texture containing 64% sand, while the sites which we did not visit averaged 48% sand based on the Web Soil Survey soil data used in the model. Based on the importance of sandy sites to *B. gracilis* distribution (Epstein et al., 1997), it is possible that habitat suitability does not necessarily exist where climatic suitability appeared highest. However, simulated C_4_ climatic suitability is high across the UGRB, and suitability will increase with climate change particularly in areas with the lowest current suitability (Figure 4). This, combined with the presence of propagules on the landscape, indicates that while C_4_ grasses are not currently common, all the ingredients for an increase in this functional type are in place and our modeling provides a glimpse into the potential future of the landscape.

### 4.3 Explaining differences in productivity changes projected at regional and local scales

Of the functional types included in our analysis, the most unexpected result was that of C_3_ perennial grasses increasing. In other similar studies, C_3_ species were found to decrease in biomass with climate change (Havrilla et al., 2023; Palmquist et al., 2018, 2021), but in our simulations of the UGRB, C_3_ grasses were projected to increase in biomass (Figures 2b,e, 4). Even though percentage change in projected C_4_ grass biomass is higher (Figure 2e), the increase in C_3_ grasses is a notable contrast to the broader‐scale studies. While these studies are important to get a sense of trends across systems, by averaging results across large regions it is possible to miss important heterogeneity at local scales.

In the UGRB, overall productivity is projected to increase as temperature and precipitation change from current levels. For all functional types except C_4_ perennial grasses, projected biomass increased by more in the UGRB than in the similar study of the Western United States by Palmquist et al. (2021). In fact, it was not until the late‐century period under RCP 8.5 that our model projected biomass decreases for any functional type (Figure S3C) or a slowing of biomass increases (Figure S3A,B). Compared to the results of the Palmquist paper in the UGRB, our results are very similar, with substantial levels of projected big sagebrush biomass and increases of the C_3_ functional group (Palmquist et al., 2021). Plant communities in different parts of sagebrush ecosystems have been shown to have differential responses to changes in climatic conditions, as increasing temperatures can push species in hot sites past their limits for optimal growth, while species in cold sites can increase growth due to the removal of temperature limitation (Kleinhesselink & Adler, 2018; Palmquist et al., 2021). Though it is possible for the photosynthetic processes of C_3_ and C_4_ species to acclimate to a wide range of temperatures (Sage & Kubien, 2007), the overall productivity increase across functional types is most likely due to the increase in temperature from climate change through the UGRB. Given the precipitation in this region, we would not expect C_4_ grasses to out produce C_3_ grasses until the temperature approaches 8°C (Epstein et al., 1997). Across scenarios, the projected temperature stayed below this except for late‐century RCP 8.5 (Table 2; Table S3), which would explain the diminishing biomass trends in that scenario (Figure S3B). Our results suggest that climate change may ameliorate the temperature limitation of growth in the relatively cold UGRB, thus resulting in an increase in biomass across functional types.

### 4.4 Limitations to modeling and field methods

In our analyses, we aimed to explore climatic suitability for different plant functional types across the UGRB under historical, current, and future climate conditions. Our methods were based on some key assumptions that could have impacted our results. First, in the simulation model STEPWAT2, we did not change the likelihood of C_4_ establishment based on the locations where we found *B. gracilis*. This was because we could not exhaustively search the entirety of the over 24,000 km^2^ UGRB, so we decided to set the establishment parameters for C_3_ and C_4_ grasses to be the same across all simulation sites. In addition, grazing is known to affect the structure and composition of plant communities (Milchunas et al., 1989), and can even alter the ratio of C_3_ and C_4_ functional types (Liang et al., 2021). However, our focus was on the potential impacts of climate suitability on plant communities, and we did not incorporate potential grazing effects in our simulations. It should also be noted that although we made efforts to account for uncertainty in future climate forecasts by assessing the impact of GCMs on outputs (Figure 3) and reporting findings using the median of multiple GCMs, it is still important to consider this uncertainty when using our results in future work.

We also acknowledge that atmospheric CO_2_ concentrations may impact future projections of plant biomass, despite being excluded from our model. We were comfortable not including CO_2_ because the impacts tend to be minimal compared to the effects of precipitation or temperature and there is not a clear pattern as to how dryland plants respond to elevated CO_2_. Some studies have found increases in atmospheric CO_2_ to benefit C_3_ plants with either negative or negligible impacts to C_4_ grasses (Ainsworth & Long, 2005; Lee et al., 2011; Luo et al., 2024). Others have determined that with long‐term exposure to high CO_2_ levels, C_4_ biomass can increase relative to C_3_ (Reich et al., 2018). Meanwhile, elevated CO_2_ can improve the water use efficiency of both functional types (Hunt et al., 1996). Given the precipitation limitation in dryland ecosystems and the generally cold temperatures of the UGRB, we determined it was most crucial to focus on changes to those factors and suggest that our results would likely be similar had CO_2_ been used as an input.

Our fieldwork was also limited by several factors. Due to the large size of the UGRB and access restrictions due to differences in land ownership, a stratified sampling of the region would not have been possible or entirely representative. Instead, we made assessments of the distribution of C_4_ grass in the form of *B. gracilis* using as many resources as we had access to. Even in our attempts to pair model results with field observations, we were unable to visit all modeled sites due to time constraints and difficulties accessing some sites due to on‐the‐ground impediments. We were also unable to assess precise soil composition requirements of *B. gracilis* in the area before our field searching, so it is possible we did not restrict our search area by soil texture as much as we could have. Despite these limitations, our results provide important information to assess the current and future plant community composition in the UGRB.

### 4.5 Implications of plant community changes on local phenology

Importantly, our results capture fine‐scale heterogeneity specific to the UGRB and provide information at a scale relevant to local land management decisions (Havrilla et al., 2023). The plant community responses to a changing climate in the UGRB have the potential to impact the structure of the entire ecosystem. In particular, changes in the relative importance of C_3_ and C_4_ grasses will alter the timing of spring green‐up. While the cool‐season C_3_ grasses tend to become green early in the spring, warm‐season C_4_ grasses generally do not reach their peak growth until later in the year (Chamaillé‐Jammes & Bond, 2010; Kalapos, 1991; Williams, 1974). Differences in the timing of greenness would impact the timing of forage availability (Chamaillé‐Jammes & Bond, 2010; Havrilla et al., 2023). This is especially important in the UGRB, due to an annual spring migration of ungulates such as pronghorn and mule deer (Kauffman et al., 2022; Sawyer et al., 2005) and the importance of livestock production in the region (Taylor et al., 2004). The current timing of the spring migration matches closely with the greening of the C_3_ vegetation on the landscape (Aikens et al., 2017). The availability of green biomass also determines the timing of all the important events in the annual cycle of the western livestock industry. Overall, climate change could result in a temporal mismatch between the productivity of plant communities and the seasonal requirements of both wildlife and cattle in the UGRB.

## 5 CONCLUSION

Our results suggest that climatic suitability for C_4_ grasses is ahead of the current distribution in the UGRB, but the presence of C_4_ propagules in the region indicates that the functional type has the potential to take advantage of suitability increases. All of the ingredients for a local C_4_ grass increase are in place, and our model provides a glimpse of the potential future plant community in the region. Importantly, because of warming temperatures, this increase in C_4_ grasses is likely to be accompanied by an increase in other functional types, including C_3_ grasses. Our work highlights the importance of considering effects which occur at the local scale in studies on the impact of climate change, and future work should assess the subsequent potential implications of these local plant community shifts.

## AUTHOR CONTRIBUTIONS

**Robert C. Anderson:** Conceptualization (equal); data curation (equal); formal analysis (equal); funding acquisition (equal); investigation (lead); methodology (equal); software (equal); visualization (lead); writing – original draft (lead); writing – review and editing (equal). **Trace E. Martyn:** Formal analysis (equal); investigation (supporting); software (lead); supervision (supporting); visualization (supporting); writing – review and editing (equal). **Rachel R. Renne:** Formal analysis (supporting); methodology (supporting); software (equal); validation (equal); writing – review and editing (equal). **Ingrid C. Burke:** Funding acquisition (supporting); project administration (supporting); resources (supporting); supervision (supporting); writing – review and editing (equal). **William K. Lauenroth:** Conceptualization (lead); funding acquisition (equal); methodology (equal); project administration (lead); resources (lead); supervision (lead); writing – original draft (supporting); writing – review and editing (equal).

## CONFLICT OF INTEREST STATEMENT

The authors declare no conflicts of interest.

## Supporting information


Data S1:

## Data Availability

All data and code used in this study are available via GitHub at this link: https://github.com/robanderson175/C3_C4_STEPWAT_Results. Code and instructions to run STEPWAT2 can be found on GitHub as well here: https://github.com/DrylandEcology/STEPWAT2

## APPENDIX A

### GCMs used in analysis

The 17 GCMs chosen from the MACA dataset included bcc‐csm1‐1, CanESM2, CCSM4, CNRM‐CM5, CSIRO‐Mk3‐6‐0, GFDL‐ESM2M, GFDL‐ESM2G, HadGEM2‐ES, HadGEM2‐CC, inmcm4, IPSL‐CM5A‐LR, IPSL‐CM5A‐MR, MIROC5, MIROC‐ESM, MIROC‐ESM‐CHEM, MRI‐CGCM3, and NorESM1‐M. Of the 20 GCMs included in MACA, we excluded three from our analysis; two because they resulted in an error when creating a weather database (BNU‐ESM and bcc‐csm1‐1‐m), and one to have an odd number of GCMs to simplify taking a median in our analysis. We removed the GCM IPSL‐CM5B‐LR because it was the worst performing of the available GCMs (Rupp et al., 2013).

### Additional STEPWAT2 inputs

The majority of inputs for simulations remained unchanged from the version of STEPWAT2 downloaded from GitHub (‘STEPWAT2’ 2021). Detailed information about these inputs and rationale for them can be found in Palmquist et al. (2018, 2021), but they generally pertain to ecological, environmental, and climatic variables of importance to each of the functional types of interest. Because the objectives of this study are to simulate the plant communities of our study region as accurately as possible, rather than investigate the impacts of climate on a large scale, it was necessary to update some of the input values. We changed the probability of cheatgrass (*Bromus tectorum*) establishment to 0.015 (Palmquist et al., 2018).

### Differences in soil texture using web soil survey

Of the 35 sites we modeled, seven only had C_3_ grasses present and had a soil composition of 61% sand and 18% clay. Sites where C_4_ grasses were present (*n* = 6) had soil composition of 64% sand and 17% clay. Sites that were not visited during fieldwork (*n* = 22) but were included in our model had soil composition of 48% sand and 24% clay.
