# Supplementary material for: Climate change and C4 and C3 grasses in a midlatitude dryland steppe
Source: Ecol Evol. 2024 Aug 1;14(8):e70103. doi: 10.1002/ece3.70103 (PMC11294577; doi:10.1002/ece3.70103)
Supplement: Supplementary file 1 — Data S1: [file ECE3-14-e70103-s001.docx]

**Supplemental Tables**

**Supplemental Table 1** Descriptive statistics of sites where Bouteloua gracilis was found prior to June 20, 2022. Matching criteria displays 5% of the range of each bioclimatic variable.

| **Bioclimatic Variable** | **Minimum** | **Mean** | **Maximum** | **Matching Criteria** |
| --- | --- | --- | --- | --- |
| **Mean Annual Temperature (°C)** | 2.50 | 2.83 | 3.02 | 0.03 |
| **Mean Temperature of Warmest Quarter (°C)** | 14.24 | 14.71 | 15.00 | 0.04 |
| **Mean Temperature of Coldest Quarter (°C)** | -9.56 | -9.24 | -9.02 | 0.03 |
| **Total Annual Precipitation (mm)** | 259.99 | 300.77 | 336.20 | 3.81 |
| **Precipitation of Warmest Quarter (mm)** | 67.74 | 70.07 | 72.63 | 0.24 |
| **Precipitation of Coldest Quarter (mm)** | 44.22 | 58.69 | 72.29 | 1.40 |
| **Sand (%)** | 47.16 | 53.51 | 58.02 | 0.54 |
| **Clay (%)** | 17.39 | 21.20 | 23.65 | 0.31 |

**Supplemental Table 2** All 125 patches of *B. gracilis* found at sites visited in the UGRB. Points are listed in no particular order. These are the patches found at 10 sites across the UGRB, with patches defined as greater than 10m apart from other patches.

| **Patch #** | **Latitude** | **Longitude** | **Patch #** | **Latitude** | **Longitude** | **Patch #** | **Latitude** | **Longitude** | **Patch #** | **Latitude** | **Longitude** | **Patch #** | **Latitude** | **Longitude** |
| --- | --- | --- | --- | --- | --- | --- | --- | --- | --- | --- | --- | --- | --- | --- |
| 1 | 42.74653003 | -109.61512 | 26 | 42.57506998 | -109.43365 | 51 | 42.59003604 | -109.468521 | 76 | 42.57199902 | -109.427606 | 101 | 42.74960803 | -109.800758 |
| 2 | 42.74525003 | -109.61679 | 27 | 42.57549 | -109.43332 | 52 | 42.589641 | -109.468223 | 77 | 42.57222801 | -109.427411 | 102 | 42.74479598 | -109.802112 |
| 3 | 42.74607003 | -109.61471 | 28 | 42.57543996 | -109.4379 | 53 | 42.58959699 | -109.467632 | 78 | 42.57243102 | -109.427108 | 103 | 42.744612 | -109.801264 |
| 4 | 42.74566996 | -109.61665 | 29 | 42.57589996 | -109.43821 | 54 | 42.58188204 | -109.431066 | 79 | 42.57260797 | -109.426769 | 104 | 42.74411797 | -109.799571 |
| 5 | 42.74577004 | -109.6188 | 30 | 42.57700997 | -109.43797 | 55 | 42.57752999 | -109.430533 | 80 | 42.57720997 | -109.43078 | 105 | 42.74371597 | -109.796717 |
| 6 | 42.77076001 | -109.77702 | 31 | 42.57703998 | -109.43756 | 56 | 42.57662902 | -109.437147 | 81 | 42.572872 | -109.426633 | 106 | 42.743548 | -109.796519 |
| 7 | 42.76925001 | -109.77521 | 32 | 42.57659004 | -109.43936 | 57 | 42.57690202 | -109.437505 | 82 | 42.57782 | -109.42971 | 107 | 42.71559804 | -109.792059 |
| 8 | 42.77020999 | -109.77621 | 33 | 42.57621001 | -109.44323 | 58 | 42.57519999 | -109.43733 | 83 | 42.57742999 | -109.43158 | 108 | 42.71564498 | -109.791035 |
| 9 | 42.76806003 | -109.77421 | 34 | 42.57552998 | -109.44277 | 59 | 42.57828402 | -109.421131 | 84 | 42.70413998 | -109.68163 | 109 | 42.712673 | -109.787435 |
| 10 | 42.75272702 | -109.780489 | 35 | 42.57656104 | -109.444215 | 60 | 42.57252297 | -109.426455 | 85 | 42.70636898 | -109.682432 | 110 | 42.71172903 | -109.78627 |
| 11 | 42.75288301 | -109.781009 | 36 | 42.57647999 | -109.44643 | 61 | 42.57286596 | -109.426336 | 86 | 42.70444298 | -109.678631 | 111 | 42.71157498 | -109.786107 |
| 12 | 42.75221899 | -109.778287 | 37 | 42.57794204 | -109.421542 | 62 | 42.57313804 | -109.425976 | 87 | 42.70445497 | -109.681833 | 112 | 42.74241904 | -109.937819 |
| 13 | 42.75276398 | -109.779987 | 38 | 42.57701098 | -109.421134 | 63 | 42.57339402 | -109.426187 | 88 | 42.70379003 | -109.68071 | 113 | 42.73085201 | -109.921857 |
| 14 | 42.57637999 | -109.43829 | 39 | 42.57439801 | -109.423842 | 64 | 42.57384304 | -109.426417 | 89 | 42.70426998 | -109.67691 | 114 | 42.55805003 | -109.450963 |
| 15 | 42.57719002 | -109.43388 | 40 | 42.57320601 | -109.425498 | 65 | 42.57383097 | -109.426686 | 90 | 42.70573002 | -109.6798 | 115 | 42.55755499 | -109.45112 |
| 16 | 42.57710997 | -109.43144 | 41 | 42.57168202 | -109.427727 | 66 | 42.57385301 | -109.426944 | 91 | 42.70688999 | -109.68145 | 116 | 42.55656098 | -109.450301 |
| 17 | 42.57631 | -109.43133 | 42 | 42.59016797 | -109.456992 | 67 | 42.57402199 | -109.427283 | 92 | 42.70466996 | -109.6829 | 117 | 42.55693498 | -109.449932 |
| 18 | 42.57723 | -109.42901 | 43 | 42.590706 | -109.460758 | 68 | 42.57427203 | -109.427488 | 93 | 42.70313004 | -109.68405 | 118 | 42.556722 | -109.44966 |
| 19 | 42.57633003 | -109.42938 | 44 | 42.59077398 | -109.467415 | 69 | 42.57748003 | -109.43102 | 94 | 42.70407401 | -109.678753 | 119 | 42.56816799 | -109.461234 |
| 20 | 42.57628997 | -109.42834 | 45 | 42.59065697 | -109.467387 | 70 | 42.57446699 | -109.426738 | 95 | 42.70604996 | -109.679078 | 120 | 42.55154701 | -109.456359 |
| 21 | 42.57693999 | -109.428595 | 46 | 42.59058497 | -109.46825 | 71 | 42.57395997 | -109.426096 | 96 | 42.745778 | -109.806425 | 121 | 42.55201497 | -109.455901 |
| 22 | 42.57750803 | -109.429285 | 47 | 42.57673999 | -109.44785 | 72 | 42.57216398 | -109.426971 | 97 | 42.74325103 | -109.796095 | 122 | 42.552222 | -109.457606 |
| 23 | 42.57499899 | -109.436493 | 48 | 42.59039797 | -109.467715 | 73 | 42.57369703 | -109.425744 | 98 | 42.74284199 | -109.794268 | 123 | 42.55221102 | -109.453837 |
| 24 | 42.57482003 | -109.43568 | 49 | 42.59034097 | -109.468094 | 74 | 42.57129 | -109.427404 | 99 | 42.74561103 | -109.805656 | 124 | 42.57106201 | -109.457181 |
| 25 | 42.57693001 | -109.44507 | 50 | 42.59032303 | -109.468388 | 75 | 42.57156098 | -109.427504 | 100 | 42.74552302 | -109.805399 | 125 | 42.571277 | -109.456875 |

**Supplemental Table 3** Temperature and precipitation medians and ranges for historical (1950 – 1980), current (1990 – 2020), mid-century (2030 – 2060), and late-century (2070 – 2099) time-periods. Values were estimated using the RCP 8.5 scenario and were calculated using a first-order Markov weather generator. Medians and ranges are across 17 GCMs and 35 sites and were averaged across the final 100 years in the simulation.

|  |  | **Temperature (°C)** | | **Precipitation (mm)** | |
| --- | --- | --- | --- | --- | --- |
| **Data Source** | **Period** | **Median** | **Range** | **Median** | **Range** |
| **MACA** | **Historical (1950-1980)** | 2.1 | 0.9 - 4.7 | 278 | 182 - 521 |
| **DayMet** | **Current (1990-2020)** | 3.1 | 2.0 - 5.3 | 303 | 182 - 614 |
| **MACA** | **Mid-Century (2030-2060)** | 7.0 | 5.4 - 9.7 | 444 | 306 - 533 |
| **MACA** | **Late-Century (2070-2099)** | 9.6 | 7.4 - 13.8 | 478 | 277 - 613 |

**Supplemental Figures**

**
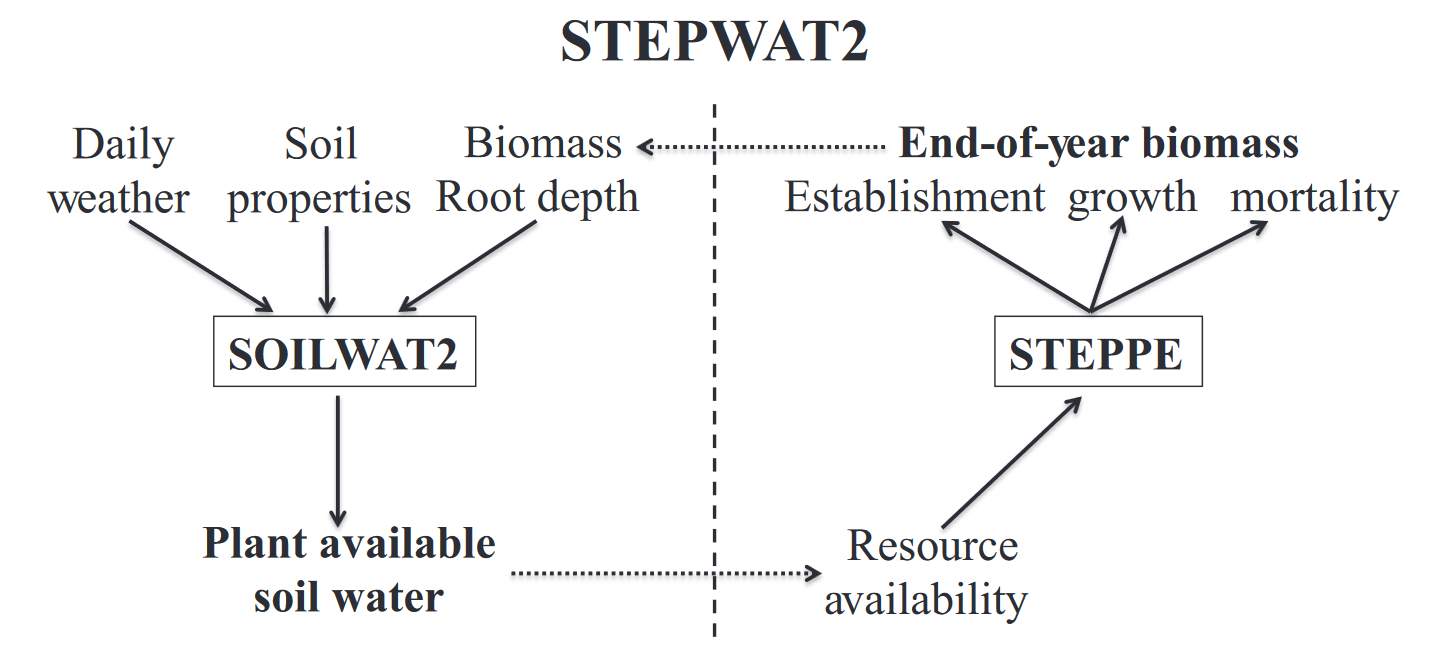
**

**Supplemental Figure 1** Conceptual diagram of the STEPWAT2 model. This diagram, and more detailed information on the inputs and mechanisms of the model, can be found in Palmquist et al. (2018).


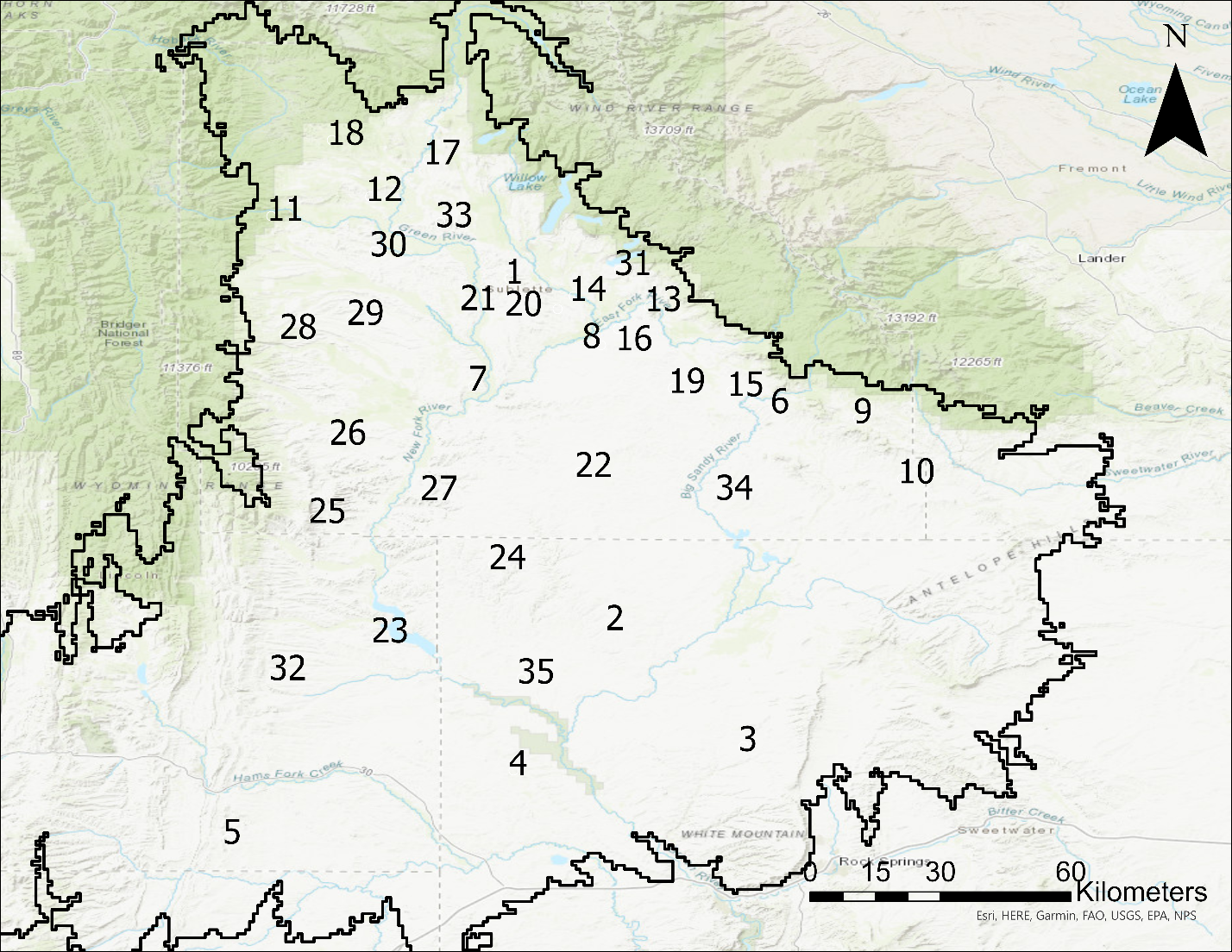


**Supplemental Figure 2** Map of sites run in STEPWAT2 model with corresponding site numbers. Reference heatmaps for site biomass results.

**
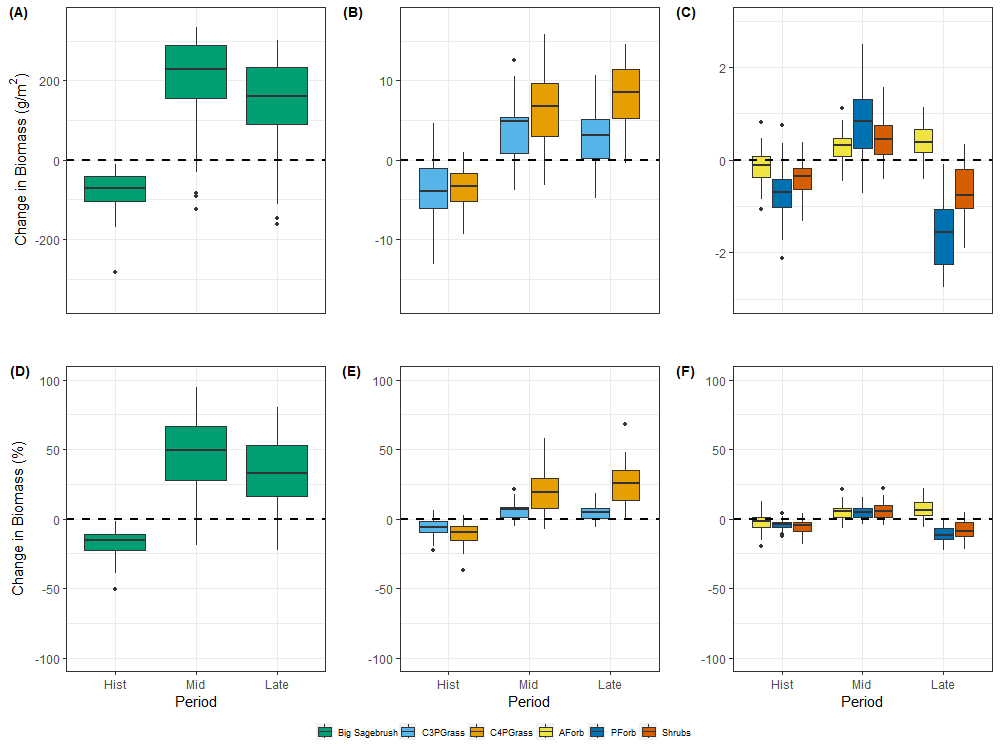
**

**Supplemental Figure 3** Changes in projected absolute biomass (gm^-2^) (A, B, & C) and percent biomass (D, E, & F) from the current (1990 – 2020) time-period (0 line) to historical (1950 – 1980), mid-century (2030 – 2060), and late-century (2070 – 2099) periods. Biomass projections were averaged across the last 100 years of model simulations, boxplots were created using values across all sites and GCMs. Here they are reported for RCP 8.5. Plots are split into major functional groups based on the magnitude of absolute projected biomass difference: (A & D) Big Sagebrush; (B & E) C_3_ and C_4_ Perennial Grass; (C & F) Annual Forbs, Perennial Forbs, Other Shrubs. Note the differences in scale of the change in each group. Each boxplot displays the median, first and third quartiles, the range of largest to smallest values, and any points on the figure are outliers (i.e. fall outside of 1.5 times the inter-quartile range).

**
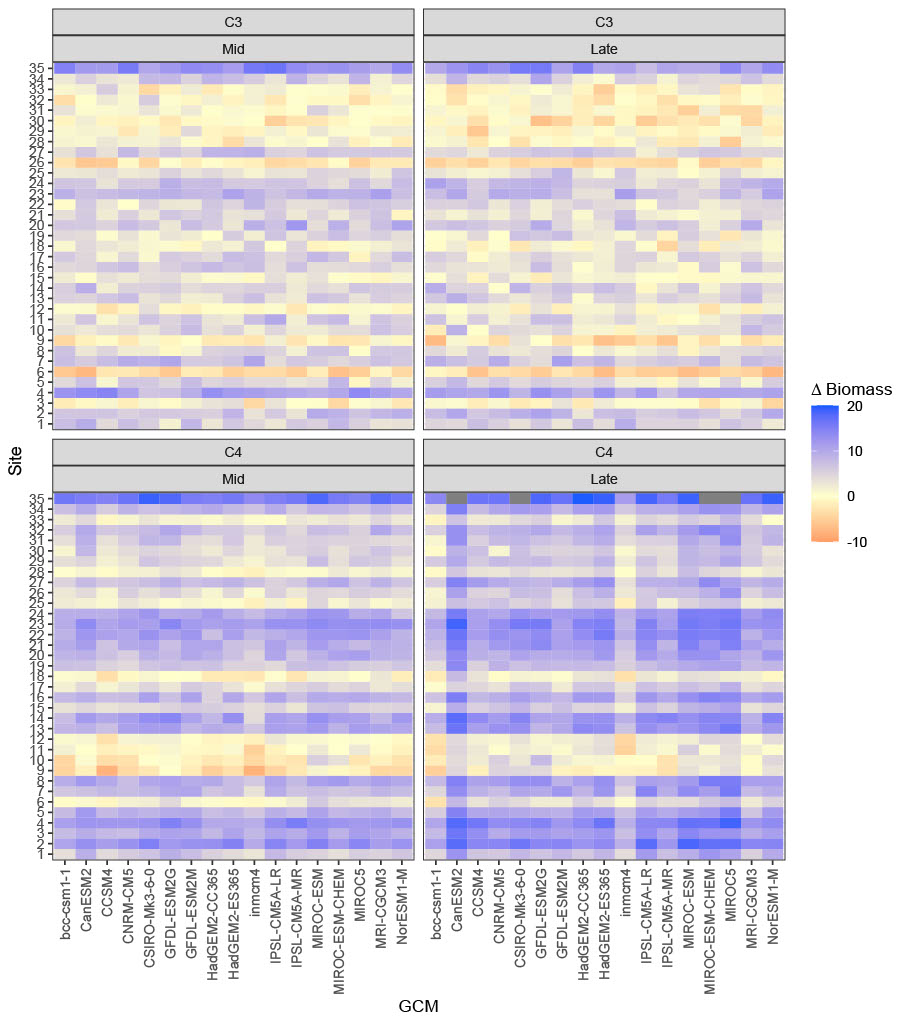
**

**Supplemental Figure 4** Heatmaps displaying the difference between biomass (gm^-2^) projections in mid-century (2030 – 2060) and late-century (2070 – 2099) time-periods relative to current (1990 – 2020) for both C_3_ and C_4_ functional groups under RCP 8.5. Projected biomass differences are reported for each combination of the 35 sites and 17 GCMs used in the STEPWAT2 simulations. Values greater than 0 indicate an increase in projected biomass from current, and values less than 0 represent a decrease in projected biomass from current values.

**
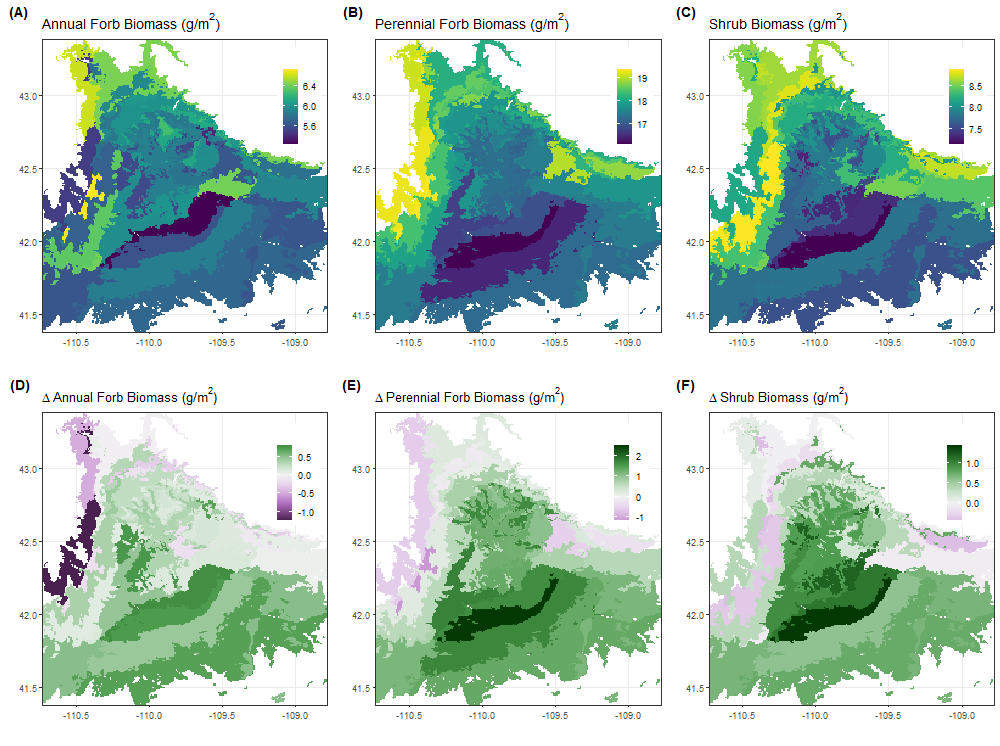
**

**Supplemental Figure 5** Maps display the Upper Green River Basin and biomass interpolated from STEPWAT2 output to create a basin-wide view of the model projections. (A, B, & C) Projected biomass (gm­^-2^) under current (1990 – 2020) climate conditions across the Basin for (A) Annual Forbs, (B) Perennial Forbs, and (C) Other Shrubs. (D, E, & F) Projected change in biomass (gm^-2^) from current to mid-century (2030 – 2060) under RCP 4.5 for (D) Annual Forbs, (E) Perennial Forbs, and (F) Other Shrubs. Values greater than 0 indicate an increase in projected biomass from current, and values less than 0 represent a decrease in projected biomass from current values. Future projections represent the median of the 17 GCMs used for each site.
